# ANCA-associated vasculitis presenting with progressive binocular manifestations: a case report and literature review

**DOI:** 10.3389/fmed.2024.1525111

**Published:** 2024-12-11

**Authors:** Lei Xi, Ying Cui, Zhilian Li

**Affiliations:** ^1^Departmen of Ophthalmology, Guangdong Provincial People's Hospital (Guangdong Academy of Medical Sciences), Southern Medical University, Guangzhou, China; ^2^Department of Nephrology, Guangdong Provincial People's Hospital (Guangdong Academy of Medical Sciences), Southern Medical University, Guangzhou, China

**Keywords:** ANCA-associated vasculitis, autoimmunity, eye, central retinal artery occlusion, keratitis

## Abstract

This report primarily describes a rare case of an elderly male patient who initially presented with central retinal artery occlusion (CRAO) in the left eye and was ultimately diagnosed with anti-neutrophil cytoplasmic antibody (ANCA) -associated vasculitis involving the eyes, gastrointestinal tract, and kidneys. However, due to irregular treatment, both eyes developed progressive ocular manifestations later. This article emphasizes the importance of actively screening for and treating underlying conditions in cases of CRAO. A comprehensive assessment of the patient’s medical history, systemic condition, and ocular examination can aid in early diagnosis, slow disease progression, and reduce mortality.

## 1 Introduction

Central retinal artery occlusion (CRAO) which is an ophthalmic emergency that occurs when the central retinal artery is blocked (1). This blockage leads to retinal ischemia, resulting in a sudden loss of vision. CRAO is often associated with systemic conditions such as carotid artery or cardiac valvular disease, hypercoagulability, atrial fibrillation, and autoimmune diseases, and it may require urgent treatment to maximize the chances of vision recovery.

According to pathophysiology, CRAO can be divided into two groups: non-arteritic, mainly due to emboli and arteritic. The arteritic category comprises less than 5% of CRAO cases and has been reported in association with giant cell arteritis (GCA), anti-neutrophil cytoplasmic antibody (ANCA) associated vasculitis (AAV) and chronic systemic autoimmune diseases (1–3).

Here, we report a unique case that initially presented with unilateral CRAO and was subsequently diagnosed as AAV. Due to poor control of ANCA, the patient later developed peripheral keratitis, corneoscleral staphylomas in the contralateral eye, and maculopathy in both eyes during follow-up.

## 2 Case presentation

The patient is a 68-year-old male who presented to the ophthalmology department with sudden, painless vision loss in the left eye for the past 24 h. He has no history of hypertension, diabetes, or other systemic diseases. Over the past 3 months, the patient had experienced unexplained weight loss of approximately 10 kg, occasionally accompanied by upper abdominal discomfort, without vomiting or diarrhea. Cardiopulmonary auscultation and abdominal palpation revealed no significant abnormalities.

**Figure 1:**
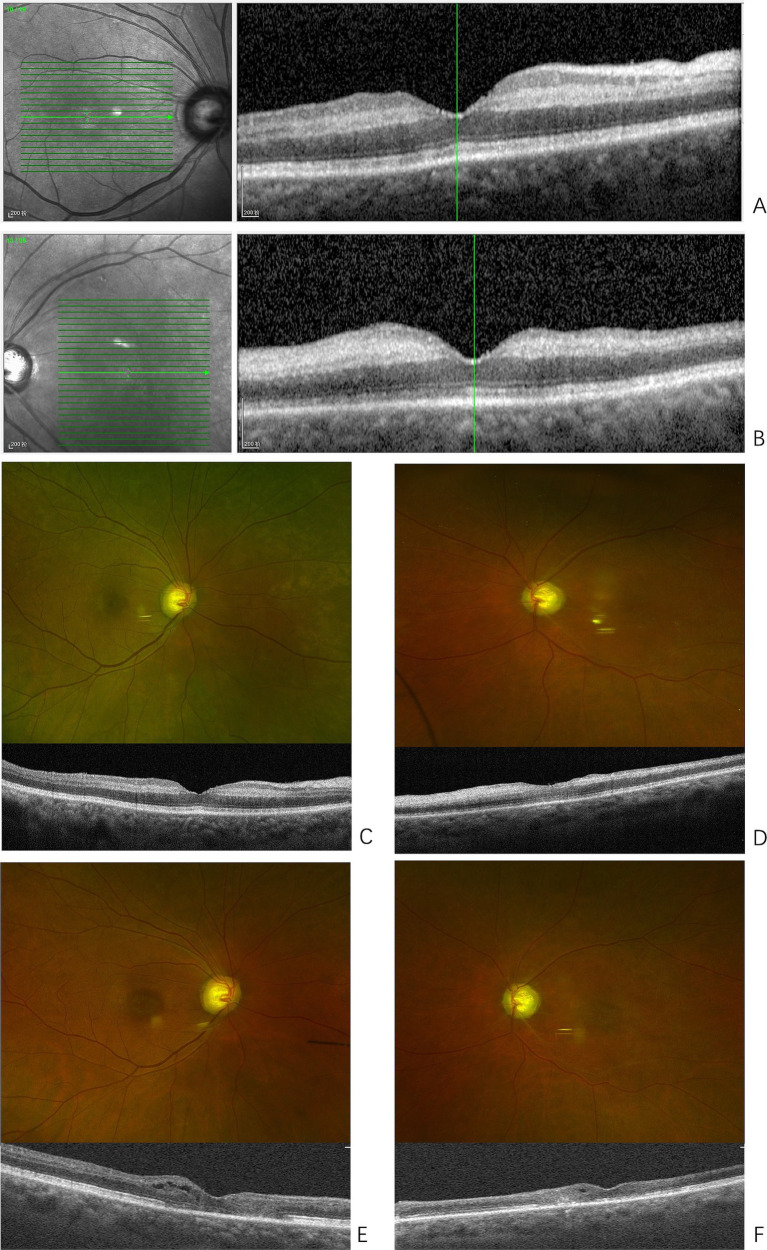
Presentations of bilateral retinopathy. **(A)** Optical coherence tomography (OCT) of the right macula at onset, shows the macular retinal thickness of the right eye is essentially normal, with edema in the inner plexiform and inner nuclear layers, and increased reflectivity. **(B)** OCT of the left macula at onset, shows enhanced reflectivity in the inner retinal layers of the macular region, with poorly defined structural layers. **(C)** Fundus photography of the right eye shows arteriovenous crossing signs in the posterior pole; OCT reveals reduced edema in the inner plexiform and inner nuclear layers of the macular region and its surroundings compared to previous findings. **(D)** Fundus photography of the left eye shows a pale optic disc, thinning of the retinal arteries in the posterior pole, and white sheaths alongside the retinal artery in the inferotemporal region of the optic disc. OCT shows reduced edema in the inner retinal layers compared to previous findings. **(E)** Fundus photography of the right eye shows an elliptical area of RPE pigment loss in the macular region; OCT reveals thinning of the inner retinal layers, with varying degrees of damage to the external limiting membrane and ellipsoid zone of photoreceptors in the central fovea and surrounding areas. Cystic cavities are observed within the retina. **(F)** Fundus photography of the left eye shows thinning of the retinal arteries in the posterior pole and white sheaths alongside the retinal artery in the inferotemporal region of the optic disc, with an elliptical area of RPE pigment loss in the macular region. OCT reveals extensive and significant thinning of the inner retinal layers, with varying degrees of damage to the external limiting membrane and ellipsoid zone of photoreceptors in the central fovea and surrounding areas. Cystic cavities are observed within the retina.

Ophthalmic examination showed no significant abnormalities in the right eye, with best corrected visual acuity (BCVA) of 20/20 and normal intraocular pressure. In the left eye, the BCVA was limited to temporal light perception only, with normal intraocular pressure. The direct pupillary light reflex was absent, while the indirect light reflex was present. Fundus examination of the left eye revealed segmented retinal artery occlusion and macular edema with a cherry-red spot, suggesting the diagnosis of left central retinal artery occlusion (CRAO). Bilateral fundus optical coherence tomography (OCT) showed normal macular thickness in the right eye with mild interlayer edema. In the left eye, the inner retinal layers in the macular region appeared poorly defined (Figures 1A,B). Emergency ophthalmic treatment was administered, including sublingual isosorbide dinitrate, ocular massage, and oral vasodilators and neurotrophic agents.

During the first 3 days of hospitalization, the patient developed dark red stools, watery diarrhea with abdominal pain, and one episode of vomiting gastric contents. His hemoglobin dropped to 85 g/L. CT scans of the chest and abdomen, along with tumor markers and gastroscopy, ruled out malignancy but suggested gastrointestinal bleeding. Concurrently, the patient’s serum creatinine rapidly increased to 456.36 μmol/L, indicating acute kidney injury (AKI), accompanied by hematuria and proteinuria (urine protein/creatinine ratio 2179.33 mg/gCr, albumin/creatinine ratio 594.81 mg/gCr). Further rheumatology workup showed elevated C-reactive protein (CRP) (116.06 mg/L) and rheumatoid factor (162.9 IU/mL). Serum cytoplasmic ANCA (c-ANCA) tested positive, and proteinase 3 (PR3) IgG antibodies were elevated (148.2 RU/mL). The patient was ultimately diagnosed with ANCA-associated vasculitis (AAV), with a BVAS score of 38 points (general condition 3 points, eye involvement 6 points, abdominal involvement 9 points, and kidney involvement 20 points).

**Figure 2:**
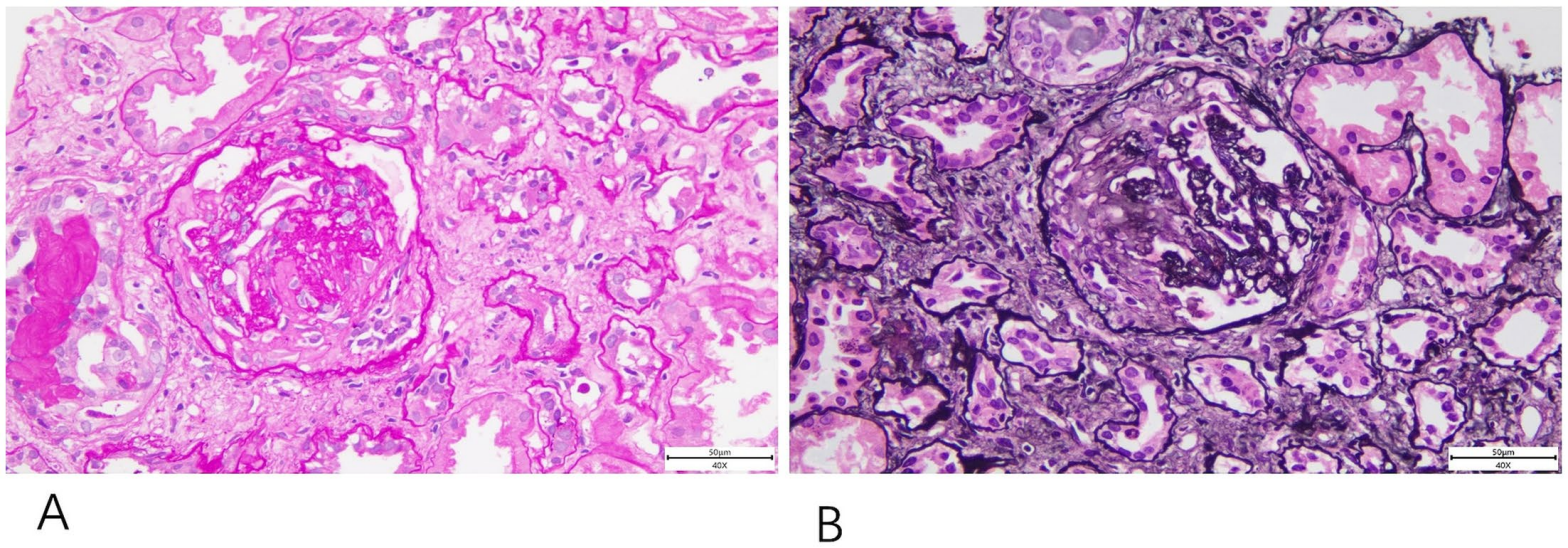
Renal biopsy histopathology. The renal biopsy shows fibrous crescent formation revealed findings consistent with ANCA-associated glomerulonephritis. **(A)** PAS staining (40×). **(B)** PASM staining (40×).

The treatment included five sessions of plasma exchange and a 3-day course of methylprednisolone pulse therapy at 500 mg/day, followed by oral glucocorticoids. Although there was no improvement in the vision of the left eye, systemic symptoms improved, serum creatinine levels significantly decreased (ranging from 119 to 180 μmol/L), anemia improved, and PR3 IgG antibodies became negative. However, before immunosuppressive therapy could be initiated, the patient developed dyspnea, accompanied by elevated inflammatory markers [hs-CRP 127.5 mg/L, procalcitonin (PCT) 2.32 ng/mL]. Chest CT, sputum culture, and sputum next generation sequencing (NGS) testing confirmed a diagnosis of pneumocystis pneumonia, complicated by respiratory failure and heart failure. Echocardiography revealed left ventricular systolic dysfunction and an apical aneurysm. After treatment, the patient’s symptoms improved. A subsequent kidney biopsy confirmed ANCA-associated glomerulonephritis (Figure 2).

Given the complications that occurred during glucocorticoid therapy, it was decided not to use cyclophosphamide or mycophenolate mofetil. The patient was then treated with azathioprine 75 mg/day, while the prednisone dose had already been tapered to 15 mg/day, and sulfamethoxazole/trimethoprim (SMX/TMP) was used to prevent Pneumocystis pneumonia. However, the patient discontinued the medication after 6 months of treatment.

**Figure 3:**
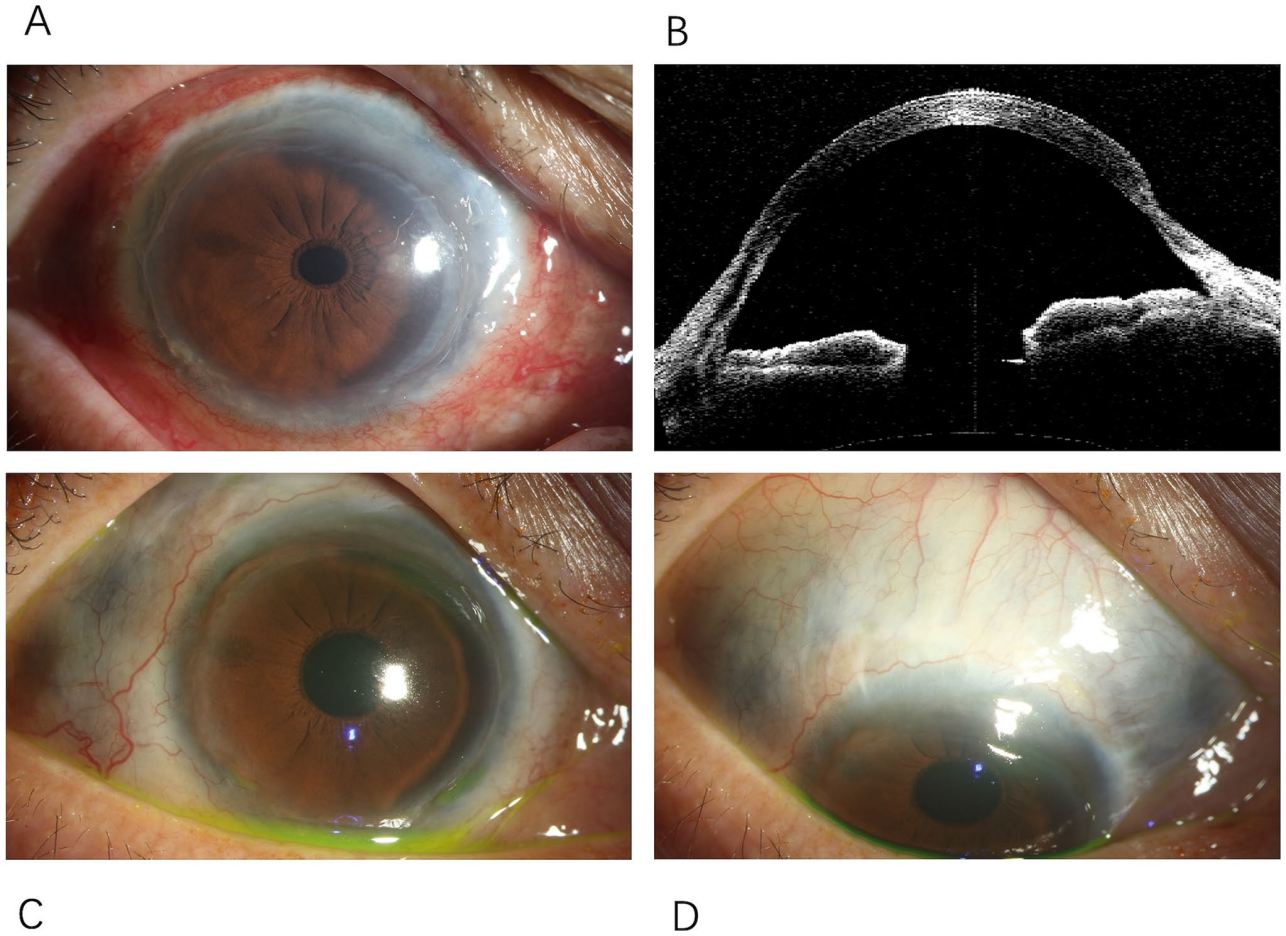
Changes in the anterior segment of the right eye. **(A)** Conjunctival mixed hyperemia in the right eye, with thinning observed around the entire cornea. **(B)** Anterior segment OCT of the right eye showing peripheral corneal thinning. **(C)** Peripheral corneal thinning observed around the entire circumference of the right cornea. **(D)** Scleral thinning in the right eye, extending from the nasal to the superotemporal region, with uveal pigmentation visible beneath.

Eight months after the onset of CRAO, the patient reported decreased vision in the right eye. BCVA was 20/200 in the right eye and counting fingers at 10 cm in the left eye. Examination of the right eye’s anterior segment revealed conjunctival hyperemia, peripheral grayish opacity around the corneal limbus, a ring ulcer, and corneal thinning (Figures 3A,B). Fundus examination of the right eye showed no significant abnormalities, and OCT indicated reduced interlayer edema in the macula compared to previous findings (Figure 1C). The left eye showed a normal cornea and anterior segment, while fundus examination revealed retinal vascular changes with a white sheath and occlusion, consistent with CRAO (Figure 1D). The diagnosis was peripheral keratitis and scleritis in the right eye, and chronic CRAO in the left eye. Local treatment included immunosuppressive and corticosteroid eye drops.

The patient subsequently experienced another episode of gastrointestinal bleeding, with positive c-ANCA and elevated PR3 antibody levels, indicating a relapse of AAV. An additional seven sessions of plasma exchange were performed, and low-dose corticosteroids combined with azathioprine were reinitiated for treatment.

Two years after the initial onset, an ophthalmic examination showed that the conjunctival hyperemia in the right eye had subsided; however, scleral thinning was observed along the corneal limbus from the nasal to the superior regions (Figures 3C,D). Macular lesions were seen in the fundus of both eyes. OCT revealed thinning of the inner retinal layers in the macular region of both eyes, with cystic cavities observed within the retina (Figures 1E,F). The final diagnoses were peripheral keratitis and scleromalacia in the right eye, maculopathy in both eyes, and chronic CRAO in the left eye. The patient ultimately passed away from massive gastrointestinal bleeding two and a half years after the initial episode.

## 3 Discussion

AAV is a group of autoimmune diseases that cause severe systemic small-vessel vasculitis characterized by the presence of ANCA in the serum (1, 2). According to the 2012 International Chapel Hill Consensus Conference, three major types of AAV have been identified: granulomatosis with polyangiitis (GPA), usually associated with upper respiratory tract symptoms, pulmonary infiltrates, and renal impairment; microscopic polyangiitis (MPA), which primarily affects the kidneys and lungs, typically without granuloma formation; and eosinophilic granulomatosis with polyangiitis (EGPA), which is usually impacting the upper respiratory tract and skin (4).

In this case, the patient presented with CRAO as the initial symptom of AAV, a rare presentation with only isolated case reports in the literature. CRAO, as an ophthalmic emergency, is typically associated with systemic conditions, such as carotid or cardiac valvular disease, hypercoagulable states, atrial fibrillation, and autoimmune diseases, and requires urgent treatment to maximize the chance of visual recovery. Arteritic CRAO accounts for less than 5% of cases and is most commonly associated with giant cell arteritis. The exact mechanism by which AAV leads to CRAO remains unclear, but underlying vasculitis and secondary localized thrombosis are considered possible causes. It is suggested that the presence of ANCA in the blood is characteristic of ischemic vasculitis, and persistent ANCA positivity may be a high-risk factor for sudden vision loss (2, 3).

While in fact, ocular involvement in AAV is not infrequent, especially in GPA. About 15% of AAV patients present with ocular symptoms at initial evaluation, and this rate reaches up to 40% in GPA patients. The incidence of ocular involvement in GPA is reported to be 2.1 to 14.4 cases per million per year (1, 2, 5). However, ocular complications in eosinophilic granulomatosis with polyangiitis (EGPA) and microscopic polyangiitis (MPA) are much lower, occurring in less than 10% of cases (6, 7). Ocular manifestations of AAV are diverse, affecting both the anterior and posterior segments of the eye, with conjunctivitis, scleritis, and blurred vision being the most common, followed by orbital pseudotumor (1, 8–14). CRAO is extremely rare in these cases (9, 13, 14).

In this case, the AAV patient initially presented with ocular symptoms, followed by sequential involvement of both eyes and the manifestation of various types of ocular symptoms. Poor control of AAV likely contributed to the further deterioration of ocular symptoms, ultimately affecting the contralateral eye. To date, no other reports have documented similar cases. Therefore, when an AAV patient exhibits unilateral ocular symptoms, it is crucial to actively treat the underlying disease while closely monitoring the contralateral eye to prevent disease progression. Additionally, the baseline macular OCT scan revealed that macular edema was primarily concentrated in the inner retinal layers, which differs from the typical CRAO presentation and should raise suspicion at the onset.

Furthermore, the patient later developed macular lesions in both eyes. Current studies suggest that AAV can lead to a variety of retinal manifestations, ranging from relatively benign cotton-wool spots (with or without intraretinal hemorrhage) to more severe vascular occlusive diseases (such as branch or central retinal artery or vein occlusions), though there is limited documentation on macular lesions (8, 9). Due to the patient’s renal impairment, we were unable to perform fundus fluorescein angiography to determine the exact cause of the macular lesions. However, they are suspected to be related to increased retinal vascular permeability, which warrants further investigation.

In summary, CRAO is often caused by systemic factors, and managing the underlying disease is essential in addition to ophthalmic treatment. AAV presents with diverse clinical symptoms, making it challenging to diagnose, and it frequently involves ocular manifestations. Without appropriate treatment, AAV carries a high mortality rate. Therefore, close monitoring of both ocular and systemic symptoms is critical for early diagnosis, assessing disease progression, and guiding treatment. A comprehensive evaluation of the patient’s medical history, systemic condition, and ocular findings is key to the early diagnosis of AAV, which can help slow disease progression and reduce mortality.

## Data Availability

The raw data supporting the conclusions of this article will be made available by the authors, without undue reservation.
